# Avoidance of medical care among American Indians with a history of cancer during the coronavirus pandemic

**DOI:** 10.3389/fpubh.2023.1265071

**Published:** 2023-11-03

**Authors:** Sixia Chen, Shirley A. James, Spencer Hall, Julie H. Dang, Janis E. Campbell, Moon S. Chen, Mark P. Doescher

**Affiliations:** ^1^Department of Biostatistics and Epidemiology, Hudson College of Public Health, University of Oklahoma Health Sciences Center, Oklahoma City, OK, United States; ^2^UC Davis Comprehensive Cancer Center, University of California, Davis, Sacramento, CA, United States; ^3^Stephenson Cancer Center, College of Medicine, University of Oklahoma, Oklahoma City, OK, United States

**Keywords:** COVID-19, coronavirus, American Indian, medical delays, appointment cancelations, cancer, disparities

## Abstract

**Objectives:**

Assess the percentage of cancer-related appointment delays, cancelations, and the unavailability of medications experienced by American Indian participants during the COVID-19 pandemic.

**Methods:**

This cross-sectional survey study was completed between October 2020 and July 2021 by 360 individuals with cancer who lived in California and Oklahoma. Binary and multivariate logistic regression analysis was completed in SAS 9.4.

**Results:**

During the initial Covid-19 pandemic, almost one-third (30%) of respondents delayed cancer-related appointments, 42% canceled cancer-related appointments, and one-quarter (24%) were unable to access prescription medications or over-the-counter medications (27%) due to COVID-19. People who underwent testing for COVID-19 were five times more likely to delay a medical appointment [adjusted odds ratio (aOR) = 5.3, 95% CI:2.4, 11.7] and people who followed three or more social distancing measures were more than six times more likely to cancel medical appointments (aOR:6.3, 95% CI:2.9, 13.9).

**Conclusion:**

This study identifies delays, cancelations, and medication inaccessibility people identifying as American Indian faced during the coronavirus pandemic. Disparities in healthcare delivery could contribute to increased morbidity and mortality rates of cancer.

## Background

The Coronavirus caused a variety of medical care delays, but perhaps none as critical as in the field of oncology. Additionally, the impact of COVID-19 was especially devastating for members of the American Indian population. Widespread fear of the pandemic and of spreading and contracting COVID-19 before the vaccine was readily available caused patients to avoid crowded places and delay critical cancer screenings (1). Patt and associates, in a study of the impact of the pandemic on senior citizens, reported that when comparing cancer screenings in 2020 to pre-covid levels, screenings decreased between 56 and 85% (2). Other studies have corroborated this finding, and note that screening delays have led to the diagnoses of serious cancer conditions at later disease stages, when they were less responsive to intervention (1–6).

In addition to screening delays, delays occurred around outpatient visits required for evaluation, intervention, and management of cancer (2–4). Inability to be in public because of exposure to COVID-19, fear of crowds due to altered immunity, and clinic staff shortages all contributed to appointment delays and cancelations (1–6). In a systematic review, Riera and associates (3) reported delays in cancer care in 38 categories, with significant decreases in cancer screenings, cancer surgeries, and radiation therapy visits. Furthermore, patients with urgent follow-up ancillary cancer screening appointments were forced to reschedule outpatient visits (3).

Other ancillary areas of cancer care were delayed for patients, resulting in undesirable consequences, including heightened mortality rates (4). Examples include chemotherapy drug delay or discontinuation, radiation or proton therapy delay, and surgical treatment delay (3–7). Many of these delays occurred in higher rates among African American patients, people of Hispanic ethnicity, and members of other racial and ethnic minority groups (8). Drug stores also reported an unprecedented increase in demand for over-the-counter medications used to treat the symptoms of COVID-19, making these medications difficult to obtain and expensive to purchase during the pandemic (9). Pain relieving drugs, nausea medication, and anti-inflammatories are all required to alleviate the symptoms, not only of cancer, but also of cancer intervention including chemotherapy and radiation therapy. Their unavailability made these symptoms unavoidable (9).

While COVID-19 associated disruptions to health care pose a risk to all patients affected by cancer, there was a disproportionate burden on the Black, Hispanic, and American Indian (AI) communities (10–13). The Centers for Disease Control and Prevention report that minority populations experienced disproportionately higher COVID-19 mortality rates when compared to Whites and other races (8). Researchers report that compared with the Black, Latino, and White populations, AI populations have a higher prevalence of socioeconomic and health-related COVID-19 risk factors (14). Moreover, AI individuals are also at higher risk for COVID-19 compared to members of the non-Hispanic White race (15–17).

Because the focus of this study was to report delays in medical care among people reporting AI race, data were collected from California and Oklahoma, two states with large AI populations (18). Data were collected during 2020–2021 and analyzed during 2022–2023. Study aims were to assess the percentage of cancer-related medical appointment *delays*, cancelations *made by patients*, unavailability of *prescription medications*, and unavailability of *over-the-counter medications* experienced by AI participants with cancer. The secondary aim was to identify factors associated with these delays, cancelations, and shortages.

## Materials and methods

### Study sample

The “Impact of COVID-19 on the Cancer Continuum Consortium” (IC-4), was established to develop and agree upon a set of core survey questions administered by 17 National Cancer Institute (NCI)-Designated Cancer Centers (Centers) across the U.S. The purpose of this survey was to assess the impact of COVID-19 on cancer prevention, control, and survivorship. This paper reports responses from a subset of survey questions administered by the University of California-Davis, Comprehensive Cancer Center (UCD), and the University of Oklahoma, Stephenson Cancer Center (OUHSC). Both Centers used a cross-sectional, non-probability-based survey design. This study was approved by the University Institutional Review Board (IRB) at each Center; in Oklahoma it was also approved by the Oklahoma City Area Indian Health Service IRB, the Choctaw Nation IRB, and the Cherokee Nation IRB. Each survey participant consented to participate in this study via telephone or by answering the consent question in the written online survey.

### Recruitment

This convenience sample included 360 AI adults. The OUHSC sample comprised AI adults diagnosed with cancer who were purposefully recruited through the Oncology Research Information Exchange Network (ORIEN) and the OU Health Sciences Center cancer registry (*n* = 328). The UCD utilized two partnering tribal health centers to recruit and administer the survey (*n* = 32). Data were collected between October 2020 and July 2021. Staff administered the 20-min surveys online, through phone interviews, and in person. Participants received gift cards as partial compensation for their time.

### Measures

#### Independent variables

Sociodemographic questions included Hispanic ethnicity (yes; no), sex (female; male), age group (18–39; 40–59; ≥60), rural vs. urban setting, marital status (single and never married; married or living together; separated, divorced or widowed), educational attainment (any post-secondary education; high school education/GED or less), income level (< $35,000; ≥ $35,000), minors residing in household (yes; no), health status (good; poor), pre-existing medical conditions other than cancer (zero; one; two; three or more), employment status before COVID-19 (full or part-time employment; unemployed or other occupation), household financial situation (have enough or more than enough; cutting back or never enough), health insurance status (yes; no), tested for COVID-19 (yes; no), time of the survey (October 2020–January 2021, February–April 2021 or May–July, 2021), number of social distancing measures followed during the pandemic (0–2; 3 or more) (social distancing measures given for participants to “check off” included wearing a face mask in public, maintaining six feet of distance between yourself and others, avoiding crowded public places, working from home instead of the office, attending conferences or large meetings online, and not inviting relatives, friends or neighbors into your home, and “other”), past 30-day close physical contact with a person who tested positive for COVID-19 (yes; no), attendance at gatherings of two or more people not living with, or regularly associated with you during the pandemic (yes; no), importance of social distancing during the pandemic (very or somewhat; a little or not), frequency of support during the pandemic (frequent; not supported).

#### Dependent variables

Four dependent variables were included in the survey. Each dependent variable was independent of each other and was binary (yes; no) in nature; “Have you ever delayed a *cancer-related* appointment due to the pandemic?” “Have you canceled a clinic, doctor, or outpatient *cancer-related* medical appointment to avoid being around others due to the pandemic?” “Have you been unable to access *cancer-related* prescription medications due to the pandemic?” and “Have you been unable to access over-the-counter medications due to the pandemic?”

### Statistical analyses

Descriptive statistics were calculated for each categorical variable. Binary associations between covariate variables and outcome were examined using the chi-square test or Fisher’s exact test when cell counts were less than five. Multivariable logistic regression was conducted, analyzing the associations between each outcome variable and the series of independent variables. Binary logistic regression modeling was first used to select the set of significant predictors for each outcome variable. After that, collinearity and interactions were examined among the selected set of significant predictors in building the final model. A stepwise model-selection procedure was used to select the final model. Tables 1, 2 present the independent variables included in the final model for each outcome variable. Adjusted odds ratios were obtained for the association between dependent (i.e., outcome) variables and independent (i.e., “predictor”) variables. Respondents with missing outcome values and predictors were excluded from bivariate and multivariate analysis. All statistical analyses were conducted using SAS^®^ 9.4 (19) with an alpha = 0.05.

**Table 1:** Sociodemographic information about study participants (*n* = 360).

Variable	Number and percentage (%)
Location	
OUHSC	328 (91.1%)
UC Davis	32 (8.9%)
Hispanic	
Hispanic	54 (20.7%)
Non-Hispanic	207 (79.3%)
Sex assigned at birth on original birth certificate	
Female	214 (59.9%)
Male	143 (40.1%)
Age Group	
18–39 years	114 (32.4%)
40–59 years	105 (29.8%)
60+ years	133 (37.8%)
Rural vs. urban setting (RUCA codes)	
Urban (RUCA codes 1–3)	212 (60.9%)
Rural (RUCA codes 4–9)	136 (39.1%)
Marital status	
Single, never been married	33 (9.2%)
Married or living together	219 (61.3%)
Separated/divorced/widowed	105 (29.4%)
Education	
Any post-secondary education	263 (73.3%)
Secondary education or less	96 (26.7%)
Income level	
<$35,000	124 (37.3%)
$35,000+	208 (62.7%)
Presence of one or more minor(s) in household	
At least one minor	147 (40.8%)
No minors	213 (59.2%)
Health status, n (%)	
Good health status	220 (61.3%)
Poor health status	139 (38.7%)
Pre-existing conditions (other than cancer)—categorical	
Zero	17 (4.7%)
One	122 (33.9%)
Two	68 (18.9%)
Three or more	153 (42.5%)
Employment status prior to pandemic	
Full- or part-time	200 (56.0%)
Unemployed or other occupation	111 (31.1%)
Retired	46 (12.9%)
Household financial situation	
Have enough or more than enough	249 (71.1%)
Cutting back or never enough	101 (28.9%)
Health insurance status	
Yes	287 (81.3%)
No	66 (18.7%)
Received testing for COVID-19	
Yes	260 (73.0%)
No	96 (27.0%)
Time period the survey was taken	
October 2020–January 2021	119 (33.1%)
February–April 2021	31 (8.6%)
May–July 2021	210 (58.3%)
How many social distancing measures did you follow?	
Zero to two	62 (19.3%)
Three or more	259 (80.7%)
Have you had past 30-day close physical contact with a person who tested positive for coronavirus or COVID-19?	
Yes	87 (25.8%)
No	250 (74.2%)
Have you attended gatherings of 2+ people?	
Yes	215 (59.7%)
No	145 (40.3%)
Importance of social distancing during COVID-19 pandemic	
Very or somewhat important	299 (84.7%)
A little or not important	54 (15.3%)
Frequency of support during COVID-19 pandemic	
Frequently supported	105 (30.6%)
Not supported	238 (69.4%)

**Table 2:** Covid-related variables and outcome measures among participants with cancer (*n* = 360).

Variables: the participant…	Had a cancer-related appointment *delayed* due to Covid-19	*Canceled* a clinic, doctor, or dental appointment because of COVID-19	Had prescription medications unavailable because of Covid-19	Had over-the-counter medications unavailable because of COVID-19
Yes	108 (30.0%)	142 (41.8%)	84 (24.1%)	90 (27.1%)
No	252 (70.0%)	198 (58.2%)	265 (75.9%)	242 (72.9%)
Hispanic				
Hispanic	18 (21.2%)	28 (25.7%)	28 (37.3%)	24 (31.2%)
Non-Hispanic	67 (78.8%)	81 (74.3%)	47 (62.7%)	53 (68.8%)
*p*-value	0.9278	0.0971	**<0.0001**	**0.0071**
Sex assigned at birth on original birth certificate				
Female	48 (44.9%)	82 (58.6%)	36 (43.9%)	45 (51.1%)
Male	59 (55.1%)	58 (41.4%)	46 (56.1%)	43 (48.9%)
*p*-value	**0.0001**	0.4601	**0.0006**	**0.0289**
Age Group				
18–39 years	52 (48.6%)	57 (41.6%)	48 (57.8%)	45 (50.6%)
40–59 years	34 (31.8%)	41 (29.9%)	29 (34.9%)	29 (32.6%)
60+ years	21 (19.6%)	39 (28.5%)	6 (7.2%)	15 (16.9%)
*p*-value	**<0.0001**	**0.0003**	**<0.0001**	**<0.0001**
Rural /Urban setting				
Urban (RUCA codes 1–3)	62 (60.2%)	85 (62.5%)	54 (67.5%)	52 (61.9%)
Rural (RUCA codes 4–9)	41 (39.8%)	51 (37.5%)	26 (32.5%)	32 (38.1%)
*p*-value	0.9014	0.6481	0.1653	0.9191
Marital status				
Single, never been married	13 (12.0%)	19 (13.5%)	12 (14.3%)	13 (14.4%)
Married or living together	72 (66.7%)	85 (60.3%)	61 (72.6%)	53 (58.9%)
Separated/divorced/widow	23 (21.3%)	37 (26.2%)	11 (13.1%)	24 (26.7%)
*p*-value	0.0598	**0.0278**	**0.0002**	0.0553
Education				
Any post-secondary education	90 (83.3%)	109 (76.8%)	71 (84.5%)	70 (77.8%)
Secondary education or less	18 (16.7%)	33 (23.2%)	13 (15.5%)	20 (22.2%)
*p*-value	**0.0063**	0.26	**0.0136**	0.1999
Income level				
<$35,000	38 (35.5%)	47 (35.6%)	21 (25.3%)	30 (34.1%)
$35,000+	69 (64.5%)	85 (64.4%)	62 (74.7%)	58 (65.9%)
*p*-value	0.6684	0.596	**0.0092**	0.3147
Presence of one or more minor(s) in household				
At least one minor	57 (52.8%)	62 (43.7%)	49 (58.3%)	43 (47.8%)
No minors	51 (47.2%)	80 (56.3%)	35 (41.7%)	47 (52.2%)
*p*-value	**0.0031**	0.1988	**0.0001**	0.0563
Self-reported Health status				
Good health status	68 (63.0%)	84 (59.6%)	58 (69.0%)	58 (64.4%)
Poor health status	40 (37.0%)	57 (40.4%)	26 (31.0%)	32 (35.6%)
*p*-value	0.6679	0.6886	0.0927	0.4048
Pre-existing conditions (other than cancer)—categorical				
Zero	2 (1.9%)	7 (4.9%)	3 (3.6%)	1 (1.1%)
One	53 (49.1%)	53 (37.3%)	45 (53.6%)	41 (45.6%)
Two	23 (21.3%)	31 (21.8%)	12 (14.3%)	19 (21.1%)
Three or more	30 (27.8%)	51 (35.9%)	24 (28.6%)	29 (32.2%)
*p*-value	**0.0001**	0.0886	**0.0002**	**0.0046**
Employment status prior to pandemic				
Full- or part-time	73 (67.6%)	87 (61.3%)	70 (83.3%)	68 (75.6%)
Unemployed/other Occ	35 (32.4%)	55 (38.7%)	14 (16.7%)	22 (24.4%)
*p*-value	**0.0038**	**0.0362**	**<0.0001**	**<0.0001**
Household financial situation				
Enough/more than enough	31 (29.3%)	100 (71.4%)	59 (70.2%)	61 (69.3%)
Cutting back/never enough	75 (70.7%)	40 (28.6%)	25 (29.8%)	27 (30.7%)
*p*-value	0.8273	0.8557	0.7117	0.6382
Health insurance coverage				
Yes	80 (74.8%)	110 (79.7%)	62 (77.5%)	70 (80.5%)
No	27 (25.2%)	28 (20.3%)	18 (22.5%)	17 (19.5%)
*p*-value	**0.0412**	0.3877	0.3708	0.641
Received testing for COVID-19				
Yes	100 (92.6%)	33 (23.7%)	74 (90.2%)	80 (89.9%)
No	8 (7.4%)	106 (76.3%)	8 (9.8%)	9 (10.1%)
*p*-value	**<0.0001**	0.1309	**<0.0001**	**<0.0001**
Time period the survey was taken				
October 2020–January 2021	25 (23.1%)	37 (26.1%)	10 (11.9%)	16 (17.8%)
February–April 2021	2 (1.9%)	13 (9.2%)	4 (4.8%)	3 (3.3%)
May–July 2021	81 (75.0%)	92 (64.8%)	70 (83.3%)	71 (78.9%)
*p*-value	**<0.0001**	**0.0121**	**<0.0001**	**<0.0001**
How many social distancing measures did you follow?				
Zero to two	15 (15.8%)	11 (8.5%)	10 (13.5%)	12 (14.5%)
Three or more	80 (84.2%)	118 (91.5%)	64 (86.5%)	71 (85.5%)
*p*-value	0.2838	**0.0001**	0.1375	0.2608
Had past 30-day close physical contact with a person who tested positive for coronavirus or COVID-19?				
Yes	43 (41.3%)	42 (31.1%)	40 (50.6%)	44 (51.2%)
No	61 (58.7%)	93 (68.9%)	39 (49.4%)	42 (48.8%)
*p*-value	**<0.0001**	**0.0121**	**<0.0001**	**<0.0001**
Have you attended gatherings of 2+ people?				
Yes	65 (60.2%)	87 (61.3%)	55 (65.5%)	58 (64.4%)
No	43 (39.8%)	55 (38.7%)	29 (34.5%)	32 (35.6%)
*p*-value	0.9175	0.7703	0.2115	0.2258
Importance of social distancing during COVID-19 pandemic				
Very/somewhat important	89 (82.4%)	124 (87.9%)	69 (82.1%)	77 (85.6%)
A little/not important	19 (17.6%)	17 (12.1%)	15 (17.9%)	13 (14.4%)
*p*-value	0.4447	0.2254	0.5088	0.9371

Bold values denote significance at the *p* < 0.05 level.

## Results

This sample consisted of 360 AI adults with a history of cancer. More participants were female (60%), were over 59 years (38%), lived in an urban setting (61%), were married (61%), had a post-secondary education (73%), had a household income level of $35,000 or above (63%), had no minors in the household (59%), reported being in good health (61%), and reported having three or more pre-existing medical conditions other than cancer (43%). Additionally, most were employed prior to the pandemic (56%), felt that they were financially stable (71%), and were covered by health insurance or a health plan (81%). At the time of the survey, most respondents had received testing for COVID-19 (73%), taking the survey May–July 2021 (58%), that is, at a time when the vaccine was widely available, followed three or more social distancing measures (81%), and had *not* had past 30-day close physical contact with a person who had tested positive for COVID-19 (74%) (Table 1).

Almost one third (30%) of participants had a delay in a cancer-related medical appointment. Binary analysis revealed males (55%) were more likely than females to experience a delay in their cancer-related appointments (*p* = 0.0001), as were those in the 18–39-year age group (49%, *p* < 0.0001). Participants who had received COVID-19 testing (93%) were also more likely to experience a delay in a cancer-related medical appointment (*p* < 0.0001) (Table 2).

Many participants (42%) canceled a clinic, doctor, or dental appointment to avoid being around others because of COVID-19. Binary analysis revealed that participants in the 18–39 year age group (42%, *p* = 0.0003), who took the survey May–July 2021 (65%, *p* = 0.0121), and who followed three or more social distancing measures (92%, *p* = 0.0001) were more likely to *cancel* a medical or dental appointment to avoid being around others because of COVID-19 (Table 2).

Respondents also had difficulty getting prescription medications related to cancer (24%), as well as over the counter medications (27%). Binary analysis revealed AI participants in the 18–39-year age group (58%, *p* < 0.0001), with past 30-day close physical contact with a person who tested positive for COVID-19 (51%, *p* < 0.0001) and who took the survey May–July 2021 (83%, *p* < 0.0001) were more likely to have had difficulty obtaining *prescription medications* because of the pandemic (Table 2). Respondents aged 18–39 years (51%, *p* < 0.0001), who took the survey May–July 2021 (79%, *p* < 0.0001), and who had close physical contact within the past 30-days with someone who had tested positive for the coronavirus (51%, p < 0.0001) were more likely to have had difficulty obtaining *over-the-counter* medications because of the pandemic (Table 2).

### The participant had a delayed cancer-related appointment due to COVID-19

Multivariate analysis revealed that after adjusting for other variables in the model, the odds of having an appointment delayed due to the pandemic were higher for those 18–39 years compared to those aged 60 years or older (aOR:2.6, 95% CI:1.4, 4.9), and were higher for those aged 40–59 years compared to those aged 60 years or older (aOR:2.1, 95% CI:1.1, 4.1). Additionally, the odds of a male having an appointment delayed due to the pandemic were higher than those of a female (aOR:2.2, 95% CI:1.3, 3.6) and odds were higher for those who received testing for COVID-19 than for those who did not (aOR:5.0, 95% CI:2.4, 11.7) (Table 3).

**Table 3:** Multivariate regression with predictors of *cancer related appointment delays*, *participant* cancelation of appointments, inaccessibility of *prescription medications*, and Inaccessibility of *over-the-counter medications* (*n* = 360)*.

Outcome variable: the participant had a *delayed* cancer-related appointment due to COVID-19		
Final number of responses for this model = 346	OR (95% CI)	*p*-value
Age (years)		
18–39 vs. 40–59	1.22 (0.68, 2.22)	0.5037
18–39 vs. 60+	**2.61 (1.37, 4.94)**	**0.0033**
40–59 vs. 60+	**2.13 (1.11, 4.09)**	**0.0235**
Sex assigned at birth on original birth certificate		
Male vs. Female	**2.16 (1.30, 3.60)**	**0.0031**
Received testing for COVID-19		
Yes vs. No	**5.30 (2.40, 11.70)**	**<0.0001**
Outcome variable: The *participant canceled* a clinic, doctor, or dental appointment to avoid being around others because of COVID-19		
Final number of responses for this model = 301	OR (95% CI)	*p*-value
Age (years)		
18–39 vs. 40–59	**2.04 (1.04, 4.01)**	**0.0386**
18–39 vs. 60+	**3.55 (1.85, 6.80)**	**0.0001**
40–59 vs. 60+	1.74 (0.96, 3.15)	0.0681
How many social distancing measures did you follow?		
Three or more vs. < three	**6.32 (2.87, 13.90)**	**<0.0001**
Time period the survey was taken		
February–April 2021 vs. October 2020-January, 2021	1.80 (0.73, 4.43)	0.2041
May–July 2021, vs. October 2020-January, 2021	**2.31 (1.30, 4.11)**	**0.0042**
February–April 2021 vs. May–July 2021	0.78 (0.32, 1.87)	0.5731
Outcome variable: The participant could not access prescription medications due to COVID-19		
Final number of responses for this model = 321	OR (95% CI)	*p*-value
Age (years)		
18–39 vs. 40–59	1.37 (0.72, 2.61)	0.3406
18–39 vs. 60+	**9.60 (3.44, 26.78)**	**<0.0001**
40–59 vs. 60+	7.02 (2.51, 19.61)	**0.0002**
Had past 30-day close physical contact with a person who tested positive for coronavirus or COVID-19		
Yes vs. No	**0.42 (0.23, 0.77)**	**0.0053**
Time the participant received the COVID-19 vaccine		
February–April 2021 vs. October 2020-January, 2021	1.32 (0.31, 5.59)	0.7038
May–July 2021, vs. October 2020-January, 2021	**3.61 (1.65, 7.91)**	**(0.0013)**
February–April 2021 vs. May–July 2021	0.37 (0.10, 1.37)	0.1356
Outcome variable: the participant could not access over-the-counter medications due to COVID-19		
Final number of responses for this model = 306	OR (95% CI)	*p*-value
Had past 30-day close physical contact with a person who tested positive for coronavirus or COVID-19		
Yes vs. No	3.11 (1.69, 5.74)	0.0003
Time period the survey was taken		
February–April 2021 vs. October 2020-January, 2021	0.48 (0.10, 2.33)	0.3613
May–July 2021, vs. October 2020-January, 2021	**2.52 (1.28, 4.97)**	**0.0073**
February–April 2021 vs. May–July 2021	**0.19 (0.04, 0.87)**	**0.0327**
Age (years)		
18–39 vs. 40–59	1.23 (0.63, 2.42)	0.5412
18–39 vs. 60+	**2.79 (1.28, 6.08)**	**0.0099**
40–59 vs. 60+	**2.26 (1.06, 4.84)**	**0.0358**

*Multivariable logistic regression was conducted, analyzing the associations between each outcome variable and the series of independent variables. Binary logistic regression modeling was first used to select the set of significant predictors for each outcome variable. Then collinearity and interactions were examined in building the final model. Stepwise model selection procedure was used to select the final model. Bold values denote significance at the *p* < 0.05 level.

### The participant canceled a clinic, doctor, or dental appointment to avoid being around others because of COVID-19

Multivariate analysis revealed that after adjusting for other variables in the model, the odds of a participant canceling an appointment to avoid being around others because of COVID-19 were higher for those 18–39 years compared to those 40–59 years (aOR:2.0, 95% CI:1.0, 4.0), and for those 18–39 years compared to those aged 60 years or older (aOR:3.6, 95% CI:1.9, 6.8). The odds of a participant who followed three or more social distancing precautions canceling an appointment to avoid being around others because of COVID-19 were higher than those who followed fewer than three social distancing precautions (aOR:6.3, 95% CI:2.9, 13.9), as well as for those who took the survey May–July 2021 compared to those who took the survey October 2020–January 2021 (aOR:2.31, 95% CI:1.3, 4.1) (Table 3).

### Inability to obtain prescription medications due to COVID-19

Multivariate analysis revealed that after adjusting for other variables in the model, the odds of having difficulty obtaining prescription medications during the pandemic were higher for those 18–39 years compared to those who were 60 years or older (aOR:9.6, 95% CI = 3.4, 26.8), were higher for those who took the survey May–July 2021 compared to those taking the survey October 2020-Jaunary 2021 (aOR:3.6, 95% CI:1.7, 7.9) and were *lower* for those who had past 30-day close physical contact with a person who tested positive for COVID-19 compared to those who did not (aOR:0.4, 95% CI = 0.2, 0.8) (Table 3).

### Difficulty obtaining over-the-counter medications during the COVID-19 pandemic

Multivariate analysis revealed that after adjusting for other variables in the model, the odds of having difficulty obtaining over-the-counter medications during the pandemic were higher for those who had 30-day contact with a person who tested positive for the coronavirus compared to those who did not (aOR:3.1, 95% CI = 1.7, 5.7), and for those who took the survey May–July 2021 compared to those who took the survey October 2020–January 2021 (aOR:2.5, 95% CI:1.3, 5.0). However, the odds were *lower* for those who took the survey from February to April 2021 compared to those who took the survey from May to July 2021 (aOR:0.2, 95% CI:0.04, 0.9). Finally, the odds of having difficulty obtaining prescription medications during the pandemic were higher for those 18–39 years compared to those aged 60 years or older (aOR:2.8, 95% CI = 1.3, 6.1), and for those 40–59 years compared to those aged 60 years or older (aOR:2.3, 95% CI:1.1, 4.8) (Table 3).

## Discussion

In this survey of American Indian participants with a history of cancer, almost one-third *delayed* a cancer-related appointment, 42% *canceled* a clinic, doctor, or dental appointment to avoid being around others due to COVID-19, and about one-quarter were unable to access *prescription* or *over-the-counter* medications due to the COVID-19 pandemic. As other studies have suggested, these delays, cancelations, and inaccessibility of medications could all contribute to long-term heightened morbidity and mortality from cancer resulting from the pandemic (2–6). California and Oklahoma are among states with the largest number of AIs (18). Our ability to collect data on a sizable number of AI individuals from these states helps to fill a gap in the collection of data on AI populations that national surveys are typically forced to avoid or neglect due to small numbers (10–13).

In this study, the most significant factor associated with a cancer-related appointment delay was being tested for COVID-19. After adjusting for both age and sex, individuals who were tested for COVID-19 were five times more likely to experience appointment delays. These results are like those discussed by Riera and associates (3); participants in their study who tested positive for COVID-19 were forced to wait for care until they tested negative, or until the isolation period passed. This study separated delays in medical care from cancelations, different from other studies that have only reported cancelations (2–6), a factor unique and important in the study of COVID-19.

Additionally, in this study more than two out of five individuals with a history of cancer canceled a clinic, doctor, or dental appointment to avoid being around others (2–6). Not surprisingly, these participants, who exercised enough caution to follow three or more social distancing precautions, were more than six times more likely to cancel one or more doctor appointments. While this finding characterizing AI participants has not been reported in the literature, this observation aligns well with several studies suggesting that social distancing during the COVID-19 pandemic was especially important for patients with chronic conditions including cancer (10–13, 15, 17). One study suggested fear of contracting COVID-19 may be at least partially responsible, particularly in patients with cancer (1).

This fear is warranted. Compared to other racial groups, the American Indian population has experienced the highest morbidity and mortality rates from COVID-19 (20–25). Musshafen et al. (15), in a study of American Indian people in Mississippi, found that the probability of death for those with the highest number of comorbidity risk factors was 69% in the AI, compared with 28% in the Black, and 25% in the non-Hispanic White population (15, 16).

Participants in our study also had difficulty obtaining prescription drugs related to their cancer intervention, as well as over-the counter medications required for physical symptoms resulting from cancer, cancer intervention, or minor medical conditions. Findings from our study were similar to others reporting chemotherapy drug delay or discontinuation because of the pandemic (7), which occurred more frequently among minorities (7). Additionally, in a survey of 68 members of the US hematology/oncology pharmacy association, 63% reported one or more chemotherapy drug shortages during the pandemic. Three quarters (75%) of the pharmacists in the study reported the need to delay chemotherapy treatment, reduce dosage, or use alternative drug regimens during the pandemic (7). Over-the-counter medications used to treat the symptoms of COVID-19 were also difficult to obtain. Drug stores reported an unprecedented increase in demand, making the most basic cold, flu, and anti-inflammatory medications difficult to obtain and expensive to purchase during the pandemic, particularly among underserved populations (9).

COVID-19 placed an immediate burden on AI communities in terms of mortality and morbidity (15–18) exacerbated by appointment delays and cancelations (1–4). One study suggests that these delays were present prior to the pandemic, although not within a subset of the population who also had a diagnosis of cancer. In 2016, Shimotsu and associates reported the odds of missed appointments were 1.8 times higher for Black/African Americans and two times higher for American Indian/Alaskan Natives and Hispanic/Latinos when compared with White non-Hispanic patients (26).

While the reasons for heightened morbidity and mortality during COVID-19 is beyond the scope of this study, accessing quality health care is difficult for people who are a part of the American Indian population, despite provision of partial health care service from the Indian Health Service (10). American Indians living within reservations often experience crowded, multigenerational, and poverty-stricken housing conditions, food insecurity, poor or no health insurance, and low incomes (10, 26). Many are employed in front-line occupations, making them more at risk of exposures (10). A clear understanding behind the reasons for gaps in quality health care for the AI population is important for preventing the heightened COVID-19 morbidity and mortality that occurred in this population from recurring.

### Limitations

This study was a convenience, cross-sectional sample in which participants’ self-reported appointment delays, cancelations, and inability to access medications. There is potential for reporting bias and non-response bias. While we collected information about the nature of missed appointments, these are not reported due to small numbers in each category. Analysis revealed that all were cancer-related appointments. Participants in the study were in various stages of cancer intervention, from new diagnoses to those requiring appointments to assess potential recurrence. Stratification by stage of intervention was also not possible due to low numbers in each category.

A control group consisting of cancer patients of other racial and ethnic groups would have allowed stratification by race, allowing us to confirm the existence of disparities in care. Conversely, a control group of patients with diagnoses other than cancer would have allowed us to stratify by diagnosis and determine whether cancer patients were treated differently or behaved differently than those with other diagnoses. These items are important for future study investigators who are forced to respond quickly to pandemic situations.

Finally, because this sample consisted of individuals with cancer recruited through the Oncology Research Information Exchange Network (ORIEN) and the OU Health Sciences Center cancer registry, as opposed to a random sample of the American Indian population, it does not represent the Choctaw or the Cherokee Nation (CN) populations accurately. Those populations are largely rural, live in a higher level of poverty, and with far less education than that reflected in Table 1. Their challenges in accessing health care are far greater than this sample of patients already receiving intervention for cancer actually reflects. Despite these limitations, this study had several strengths. First, this is the largest study to investigate the influence of Covid-19 on delays in medical care among AI adults with cancer, specifically associated with the Covid-19 pandemic. Second, the survey questions queried delays or postponements in medical care separately from the cancelations. Third, the delays and cancelations reported in this study were initiated by the patient and not by the medical clinic. This provides a clearer picture of the impact of the virus on the patients receiving healthcare during the pandemic, as opposed to clinic-related shortages.

### Public health implications

COVID-19 placed an immediate burden on AI communities in terms of mortality and morbidity (15–18) exacerbated by appointment delays and cancelations (1–4). While we hope to never experience another pandemic, lessons learned suggest that we must overcome institutional barriers causing treatment delays and explore alternative options such as telehealth visits to ensure continuation of care, especially to those vulnerable to racial disparity.

## Data availability statement

The raw data supporting the conclusions of this article will be made available by the authors, without undue reservation.

## Ethics statement

The studies involving humans were approved by the University of Oklahoma Health Sciences Center IRB, University of California-Davis IRB, Oklahoma City Area Indian Health Service IRB, the Choctaw Nation IRB and the Cherokee Nation IRB. The studies were conducted in accordance with the local legislation and institutional requirements. The participants provided their written informed consent to participate in this study.

## Author contributions

SC: Writing – review & editing, Conceptualization, Data curation, Formal analysis, Investigation, Methodology, Project administration. SJ: Writing – review & editing, Writing – original draft. SH: Data curation, Formal analysis, Writing – review & editing. JD: Conceptualization, Methodology, Supervision, Validation, Writing – review & editing. JC: Conceptualization, Investigation, Methodology, Supervision, Writing – review & editing. MC: Conceptualization, Data curation, Formal analysis, Funding acquisition, Investigation, Methodology, Project administration, Resources, Supervision, Validation, Visualization, Writing – review & editing. MD: Conceptualization, Data curation, Formal analysis, Funding acquisition, Investigation, Methodology, Project administration, Resources, Supervision, Validation, Visualization, Writing – review & editing.
